# Respiratory Diseases, Malaria and Leishmaniasis: Temporal and Spatial Association with Fire Occurrences from Knowledge Discovery and Data Mining

**DOI:** 10.3390/ijerph17103718

**Published:** 2020-05-25

**Authors:** Lucas Schroeder, Mauricio Roberto Veronez, Eniuce Menezes de Souza, Diego Brum, Luiz Gonzaga, Vinicius Francisco Rofatto

**Affiliations:** 1 X|Reality and Geoinformatics Lab., Vale do Rio dos Sinos University, São Leopoldo 93022-750, Brazil; schroederlucas@hotmail.com (L.S.); veronez@unisinos.br (M.R.V.); diebrum@unisinos.br (D.B.); lgonzaga@unisinos.br (L.G.); 2 Department of Statistics, State University of Maringá, Maringá 87020-900, Brazil; 3 Department of Geography, Federal University of Uberlândia, Uberlândia 38408-100, Brazil; vinicius.rofatto@ufu.br

**Keywords:** health, fire, big data, Data Mining, Knowledge Discovery from Databases, machine learning

## Abstract

The relationship between the fires occurrences and diseases is an essential issue for making public health policy and environment protecting strategy. Thanks to the Internet, today, we have a huge amount of health data and fire occurrence reports at our disposal. The challenge, therefore, is how to deal with 4 Vs (volume, variety, velocity and veracity) associated with these data. To overcome this problem, in this paper, we propose a method that combines techniques based on Data Mining and Knowledge Discovery from Databases (KDD) to discover spatial and temporal association between diseases and the fire occurrences. Here, the case study was addressed to Malaria, Leishmaniasis and respiratory diseases in Brazil. Instead of losing a lot of time verifying the consistency of the database, the proposed method uses Decision Tree, a machine learning-based supervised classification, to perform a fast management and extract only relevant and strategic information, with the knowledge of how reliable the database is. Namely, States, Biomes and period of the year (months) with the highest rate of fires could be identified with great success rates and in few seconds. Then, the K-means, an unsupervised learning algorithms that solves the well-known clustering problem, is employed to identify the groups of cities where the fire occurrences is more expressive. Finally, the steps associated with KDD is perfomed to extract useful information from mined data. In that case, Spearman’s rank correlation coefficient, a nonparametric measure of rank correlation, is computed to infer the statistical dependence between fire occurrences and those diseases. Moreover, maps are also generated to represent the distribution of the mined data. From the results, it was possible to identify that each region showed a susceptible behaviour to some disease as well as some degree of correlation with fire outbreak, mainly in the drought period.

## 1. Introduction

Fire is one of the essential components for the development and maintenance of some economical resources. However, humans have been made extensive use of fire to clear forests and vegetation and to prepare and maintain land for agriculture [1,2]. Between 2003 and 2012, about 67 million hectares of forest were burned, mostly in Africa and South America [3]. This deforestation fires practice causes disturbance in the ecosystem, which affects human health. In Brazil, for example, deforestation have transformed the forest fringes and created abundant larval breeding sites for *Anopheles darlingi*, the dominant malaria vector in Brazil [4]. Some researchers have also found an increase in leishmaniasis and malaria cases due to ecosystem disturbances that led to more human-mosquito interactions [5,6,7]. In addition, the increased fire impacts on human respiratory system due to enhance of aerosol emissions with degradation in air quality. This is more evident in children under-fiver in municipalities highly exposed drought [8]. As point out by the same authors, aerosol was the primary driver of hospitalisations in drought affected municipalities during 2005 in Brazilian Amazon.

Deforestation rates from the National Institute for Space Research (Portuguese: Instituto Nacional de Pesquisas Espaciais—INPE) together hospitalisations from the Brazilian Health System (SUS) provide the opportunity to study the spatial and temporal association between those diseases (malaria, leishmaniasis and respiratory) and the region where the fire outbreak is more expressive. Brazil’s National Institute for Space Research has been monitoring fire and deforestation in the Amazon via satellite since 1988. These data have been important for measuring the magnitude and spatial distribution of forest degradation and their relationship with diseases [9,10,11,12].

However, the big challenge is how to compile these massive data efficiently because the increasing volume, variety and velocity of digital data easily overload conventional analysis techniques. In this case, raises the need of specific approaches of artificial intelligence to manipulate and guaranty reliability/veracity regarding such amount of data, such as Knowledge Discovery from Databases (KDD) [13]. One of the steps in KDD refers to statistical methods of data mining for fast extraction of useful information from massive and complex spatial/geographic and temporal data [14].

Thus, to deal with 4 Vs (volume, variety, velocity and veracity) associated with these data we proposed a method that combines two approaches based on Data Mining and KDD. First, the Decision Tree, a machine learning-based supervised classification, to extract only relevant and strategic information, namely States, Biomes and period of the year (months) with the highest rate of fires. Second, the K-means, an unsupervised learning algorithms that solves the well-known clustering problem, is employed to identify the groups of cities where the fire occurrences is more expressive. Finally, the steps associated with KDD is perfomed to extract useful information from mined data to discover spatial and temporal association between diseases and the fire occurrences. In that case, Spearman’s rank correlation coefficient, a nonparametric measure of rank correlation, is computed to infer the statistical temporal dependence between fire occurrences and those diseases. Moreover, maps are also generated to represent the distribution of the mined data. Hence, with this proposal we are going to answer these two questions: “When and Where the highest fire rate occur? and “Is there a spatial and/or temporal correlation between region with high fire rates and Malaria, Leishmaniasis and/or Respiratory diseases?”.

The rest of the paper is organised as follows. In Section 2 we briefly show the reason for investigating malaria, leishmaniasis, and respiratory diseases. Section 3 describes the available data and the proposed method in details. Results and discussions are displayed in Section 4. Finally, final remarks are provided in Section 5.

## 2. Malaria, Leishmaniasis and Respiratory Diseases

In addition to environmental damage with flora and fauna destruction mainly because changes in habitat and climate [15], fire outbreak also causes social, economical, and health damage to people [16]. Besides harmful effects on the respiratory system, deforestation from fires also generates significant increases in other diseases that often go unnoticed like Malaria and Leishmaniosis [17,18,19].

Leishmaniasis is becoming an important subject in the world and is increasing every day. The World Health Organization (WHO) estimated that 350 million people are at risk of becoming infected and that 12 million people are already infected. Leishmaniasis has been identified in 12 countries in Latin America, with 90% of the cases in Brazil, occurring in almost all Brazilian states. As a control strategy, insecticides and appropriate treatments are used, but they are done in isolation, thus determining a need for reevaluations in the regional forms of control of leishmaniasis [20]. Environmental changes caused by humans have modified the epidemiological profile of leishmaniasis in areas where transmission is related to wildlife, as well as in areas where transmission is in rural or urban areas. In such areas, transmission depends on the adaptation of certain species of mosquitoes that can be generated through environmental changes [21].

Malaria remains a major public health problem and has increased significantly through changes in environmental characteristics [17]. In 2007, almost 100% of malaria cases in Brazil, occurred in the Amazon, totaling about 460,000 cases of the disease [22]. This disease is a highly discussed issue in the world network, and this is one of the major parasitic concerns due to the high rate of hospital morbidity, mainly in the Amazon Region [23]. However, there is a lack of firm assumptions about where the transmission occurs. Studies with the best spatial and temporal resolution are necessary to better understand the transmission mechanisms of the disease [24].

Related to respiratory diseases, according to official Organizations for health protection, wildfire emissions can have acute or long term health implications on the exposed populations [25]. The burning of biomass produces gaseous pollutants and emissions of fine particulate matter, which produce detrimental effects on the respiratory system [26]. Among the major components of wildfire smoke that can affect ambient air quality, is important to mention the fine particles PM2.5 and PM10, whose effects are expected to further increase when their concentrations get above air quality standards [25].

## 3. Material and Methods

**Figure 1:**
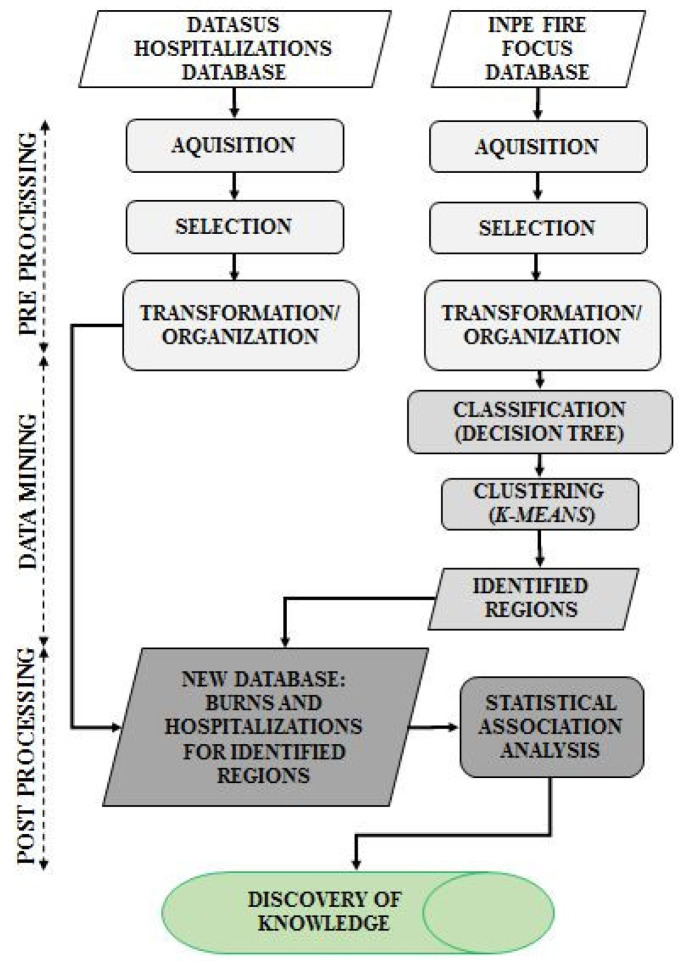
Flowchart of the the involved steps from pre-processing, classification (decision tree), clustering (K-means), post-processing until the knowledge discovery (KDD).

As point out by [14], spatial data mining is not a push button task. Actually, it requires meticulous selection, pre-processing, and transformation/organization of the data in order to ensure meaningful analysis and results. In addition, it also demands efficient computational algorithms to process large data sets and effective visualization approaches to present and explore complex patterns. Thus, the involved steps from pre-processing, classification (decision tree), clustering (K-means), post-processing until the knowledge discovery are showed in Figure 1 while the characterizations of each step will be described in the following sections.

### 3.1. Pre-processing

The three pre-processing steps in the flowchart of Figure 1 are, actually, the preparation of the data for mining [27]. They are going to be described for the both databases of interest in this study.

#### 3.1.1. Forest fires Database

The fire focus database was obtained from INPE that uses remote sensing (satellite images) to distinguish between green vegetation and burned surfaces [28]. According to [29], the reference satellite product makes use of active fire observations from several sensors and depends on a burn-sensitive Vegetation Index. As for the location of the daily fires, the information is indicated by the Moderate Resolution Imaging Spectroradiometer (MODIS) sensor, where the foci are represented by 1 pixel, with a spatial resolution of 1 km [11]. The database available by INPE is updated every three hours throughout Latin America and contains information on geographic coordinates of the fire outbreaks, biome, estimates of smoke concentration in the air, fire risk, expected precipitation, number of days without rain, date, time, and city of the observed focus [30]. The next step in the pre-processing is to select the required data. However, gathering this information into a database is not always an easy task, as this process may involve poor quality data analysis [31]. The forthcoming data mining step can help verifying the quality of the selected data. In the selection step of this research, considering the evaluated period (2011–2018), the year with the highest occurrence of fires, 2017 with 260,138 active fire records, was selected. Preprocessing also includes rearranging/transforming raw and nonstandard data into data of simple formats, such as spreadsheets and tables. This step is considered to be one of the most laborious and time consuming before applying Data Mining, as it includes techniques for obtaining, merging, cleaning, removing noise, and duplicating data [32]. In this paper, the data from each state was obtained and merged. Furthermore, variables with too much missing values were ignored. This was the case of the smoke concentration in the air (more than 90% of missing values). All the other available aforementioned variables were completely registered and then considered in this paper.

#### 3.1.2. Health Database: Malaria, Leishmaniasis and Respiratory Disease Hospitalizations

In Brazil, the Department of Information Technology of the Unified Health System (SUS) has a platform that makes data from public health systems available via internet (DATASUS, 2018). The causes of hospitalization are registered following the International Classification of Diseases, considering its 10th revision (ICD-10). The hospitalizations related to all respiratory diseases (codes J00-J99), malaria and leishmaniasis (B50-B55) were considered. In the selection step, although several variables of the patients are registered in the database, in this study we considered the city of residence, age, and details of the hospitalization (CID, day of hospitalization, etc.).

### 3.2. Data Mining

The main objective of Data Mining is the extraction of standards/patterns and relationships among variables in large databases [33,34]. In data mining approaches, one of the main advantages is the possibility of building interpretable statistical learning models providing qualitative and quantitative understanding of the relationship among the features of interest [35].

These models can be built for either supervised (outcome variable is predicted based on input variables) or unsupervised learning (look for associations among input variables without an outcome measure). First, a supervised classification learning was carried out to identify only relevant and strategic attributes (states, biomes, period of the year) associated with high rates of fires. Then, an unsupervised learning was performed from clustering methods to identify critical regions (group of cities) for posterior temporal analysis/association with diseases. Following subsections describe these methods.

#### 3.2.1. (a) Classification: Decision Trees

Regression or Decision trees can be considered an ideal base learner for classification [35]. It is a flexible predictive model with an hierarchical nature able to identify a relevant attribute and subdivide the others into subsets characterized by its class [35,36]. It can be depicted in graphs of easy interpretation, in which there is a single root node and a collection of resulting axis and nodes, representing the relationships between the attributes [33,37]. Is it possible to implement the decision tree method through fast algorithms. As usually multivariables are available, the decision tree J48, which is the implementation of algorithm ID3 (Iterative Dichotomiser 3), is promise because it accounts, e.g., for missing values, decision trees pruning, continuous attribute value ranges, and derivation of rules. We used the Waikato Environment for the analysis of knowledge (WEKA) tool [38], where J48 is an open source JAVA implementation of the C4.5 algorithm [38,39,40]. Weka software contains some incremental algorithms that can be used to process very large data sets and allows the user to specify a data stream by connecting preprocessing components, algorithm learning, evaluation methods, and visualization modules [38]. Thus, biomes, states, months, and periods with high rates of fires could be identified and classified in an agile way. Although these variables are available in the dataset, classifying each one from the others, allows us to verify the classification performance and the reliability of the information in INPE’s dataset. The reader could ask why not just compute frequency tables to reach this information? The key point here is that is common the management of geographic data of poor quality or with a lot of noise. Several analyses and investigations are needed to check duplicates, outliers, and inconsistencies of several kinds. Unfortunately, mainly for large datasets, we can not just believe in the database and perform analysis of counts or densities, which would be certainly the first natural and usual descriptive analysis. Of course, using a specific platform because even simple frequency tables for a relatively large amount of data are not possible to be handled in tradicional softwares such as Excel. Counting just frequencies regarding to e.g., biomes and states, we can not verify that different observations can be registered with the same coordinates but different biomes or states. These inconsistencies are due to the variability and/or limitation of the measurement processes and may be negligible to some regions, but they can be really serious for others. The user can ignore that and reach e.g., wrong classifications in a posterior step of Geographic Information System spatial evaluations or have a lot of work crossing information of several variables and double checking the database. On the other hand, instead of losing a lot of time to verify the consistency of a database, with decision tree it is possible to identify important variables and perform a fast management and extraction of useful information of a database with the knowledge of how reliable the dataset is. Besides identifying the variables, it is possible to know their order of importance.

Since Decision Tree is a supervised learning method, the dataset was splitted into 70% for training and 30% for test. The training dataset are the sample of data used to fit the model removing the respective variable that is being predict. The test set was used only to assess the performance of the Decision Tree classifier.

To evaluate the classification, an error matrix (also called confusion matrix) represents the amount of information analyzed and the class that are assigned, from which the Global Accuracy (GA) can be calculated as

(1) $$GA=100\sum_{i=1}^{r}\frac{x_{ii}}{m}$$

where *r* is the number of rows of the matrix, $x_{ii}$ are the values stored in row *i* column *i*, and *m* is the quantity of analyzed data [41]. Kappa statistic was also analyzed to show how far the observations are dispersed from the expected [42].

#### 3.2.2. (b) Clustering

After the identification of states, biomes and critical periods for fires in the classification stage, there is the need of identifying within the states, groups of cities that present significant amounts of fires, to finally be able of looking for associations with hospitalization data (by place of residence) available by DATASUS.

Grouping or clustering, as is best known, is a method of grouping rows of data that share similar trends and patterns into distinct groups. Clustering studies do not have an independent variable as in the classification process, what makes the clustering an unsupervised grouping method [43].

Data clustering is directly based on similarity calculations [44], which are key in the clustering scenario. They are the input for K-means. Although there are other approaches to identify clusters, K-means cluster detection lies at the heart of spatial data mining (point event data) as a statistical tradition of classification which assigns point events to a spatial segmentation. Thus, K-means is one way to achieve our goal by performing data aggregation at central nodes.

To determine whether a set of points, given by the curvilinear geodetic coordinates of fire occurrences, is similar/close enough to be considered a cluster or not, a distance metrics between points is required. The measure of distance between (x,y) tells how far the points *x* and *y* are. Many distance functions can be used as measures of similarity. Each of these distances comes with its own geometry, which implies in the formed clusters [37]. The commonly used distance measures are Manhattan Distance, Euclidean Distance, and Minkowski Distance [45]. In this work, the Euclidean Distance Equation was used

(2) $$d\left(x,y\right)=\sqrt{\sum_{j=1}^{n}{\left(x_{j}-y_{j}\right)}^{2}}$$

where $x_{j}$ and $y_{j}$ are the geographic coordinates (latitude and longitude) and *n* is the number of fire occurrences.

The basic idea behind partitioning methods, such as K-means clustering, is to define “*k*” clusters such that the total intra-cluster variance (or total within-cluster sum of square—WCSS) is minimized. The method will create centroids $c_{i}$ in each grouping at random and then the iterations will be done in order to adjust the centroid [33]. Conceptually, the centroid of a cluster is its center point, computed usually by the average although other measures may be used. The quality of the clustering can be measured by the square of the distance between the sample points in each cluster and the centroid, which is the WCSS [32]:

(3) $$WCSS=\sum_{i=1}^{k}\sum_{j=1}^{n_{j}}d{\left(x_{j},c_{i}\right)}^{2}.$$

Besides a performance indicator, changing the k-values in WCSS (Equation (3)) is possible to find the optimal number of clusters in a set of observations [32]. This approach is called Elbow method and its main purpose is to try to make the resulting “k” clusters as compact and separate as possible. In practice, the k value can be better determined by plotting the WCSS curve in function of *k* and by finding the inflection point down (when adding another cluster does not improve much the total WCSS).

Summarizing, the step-by-step method is applied as follows [33]:

- determine the number of *k*, that is, the number of centroids and clusters that will be created;
- calculate the distance of each sample observation to the centroid;
- reposition the sample observation for the group whose distance to the centroid is smaller;
- recalculate the new position of the centroid within its group;
- repeat the iterations until the centroid does not change its position;

With the aforementioned K-means cluster technique, we identified the groups of coordinates where the fire occurrences are concentrated. Each group represents fire occurrences that are close to each other (centroid), since the Euclidean distance is the measure of similarity among the coordinates. If we had considered a simple method such as just counting of fire by municipalities, just the municipalities identified with more fire occurrences would be identified. For instance, if the fire occurrences are close to the edge of a municipality, the fire consequences may be even more intense to the neighborhood depending on the proximity to some city or the size of the municipality. To minimize this problem, we considered all the municipalities that had some fire occurrence in the identified cluster.

After the data mining classification and clustering, the association of burn clusters with hospitalizations due to respiratory diseases, Malaria and Leishmaniosis to generate Knowledge Discovery can be performed by integrating these databases. This is going to be part of the post-processing step, which is presented in the next section.

### 3.3. Post-Processing

According to [32], post-processing only ensures that useful data are incorporated into the decision-making and KDD process. Visualization is an example of post-processing, as it attempts to portray the data obtained in Data Mining in a way that the human being can visualize, exploit, and better understand the obtained results [13,46]. Thus, statistical analysis and graphic tools will be used integrating the results from data mining (classification and clustering) with health data to investigate relationships between the burns and diseases.

To illustrate this analysis, the 3 states with the largest occurrences of fires were considered. For each state, the clusters of cities with hotspots of fire occurrences will be identified, and the hospitalizations due to respiratory diseases, Malaria, and Leishmaniosis were accounted for the cities in these clusters. Considering that burnings may influence hospitalizations due to respiratory diseases in a short period of time, the temporal association analysis in this case is going to be performed considering the results of the period of the year with more occurrences of burnings. Furthermore, considering that children is the most affected group regarding to respiratory diseases, this analysis was performed for children 0–4 years old. On the other hand, considering the incubation period of malaria is not necessarily short, it is difficult to restrict the analysis to some period of the year. Thus, all occurrences during the year were considered as well as all hospitalizations without restriction by age. It is important to highlight, the hospitalizations were considered by place of residence, because the objective is to identify the relationship between burnings and their impacts in a regional way.

For the analysis of the relationship between fire focus with diseases, the annual time series of the aggregated occurrences in the clusters of each state were analysed from 2013 to 2018. The time series of hospitalizations were built as annual rates in 100,000 inhabitants:

(4) $$\mathrm{Hospitalization}\;\mathrm{rate}=\frac{\mathrm{Total}\;\mathrm{of}\;\mathrm{hospitaliation}\;\mathrm{cases}}{\mathrm{Total}\;\mathrm{population}}.$$

The correlation coefficient of Spearman, $r_{s}$, was computed and the association was evaluated as very weak if $0\leq |r_{s}|\leq 0.19$, weak if $0.2\leq |r_{s}|\leq 0.39$, moderate if $0.4\leq |r_{s}|\leq 0.59$, strong if $0.60\leq |r_{s}|\leq 0.79$, and very strong if $0.8\leq |r_{s}|\leq 1$ [47].

Considering young children are in general the most affected group, we built and evaluated the time series for 0-4-year-old children and also in the period of more occurrences of fires (July to December). All but decision tree classification were implemented in Python. Pandas, which is a Python library that provides high-level data analysis frameworks, was also used. One of the greatest qualities of this library is the ability to process complex data with few commands and with high speed [48]. Furthermore, considering that the task of clustering is a recognized challenging conceptually and computationally, sklearning was used [49]. Considering that dealing with large datasets can be time consuming, the time spent in this classification step is going to be presented, which was reached using a computer with an Intel® Core ™ I7 Processor, Windows 10 Operating System, and 8 GB of RAM.

## 4. Results and Discussion

### 4.1. Classification

In the classification stage, biomes, states, months, and periods with high rates of fires were predicted/classified from the fire database.

#### 4.1.1. Biome

For biome classification, the geographic coordinates of fire outbreaks, fire risk, expected precipitation, number of days without rain, identification of States and Cities were used in the decision tree classification method. The error matrix per biome of testing dataset is presented in Table 1 showing the most striking biomes in the identified red rectangle.

The classification by biome generated an overall accuracy of 98.86%. The main diagonal of the matrix identified in bold represents the correct classifications when testing the trained model. To show how far the observations are dispersed from the expected, the Kappa statistic was also analyzed, which resulted in a value of 0.98, this can be considered an excellent result [42]. Regarding to the processing time, the classification method using the training base (70% of the data) required 8.16 s to construct a model to identify which biomes had a higher incidence of fires. The time required to test the model on 30% of the data took just 0.69 s.

We also carried out the classification using the whole dataset to confirm which biomes in 2017 had the largest number of fires. From the results, the Amazon biome was confirmed as the precursor of the list, with 132,390 fires and the Cerrado with 85,605 fires observed. These two Biomes account for 83.80% of all fire outbreaks observed in the year 2017. Also, we can observe from the testing dataset in Table 1, where 39,641 and 25,637 forest fires were observed and classified correctly for Amazon and Cerrado, respectively. These numbers are compatible with the literature [11,50]. In the study conducted by Araújo, Ferreira and Arantes (2012) [50], through the analysis of fires in the period between 2002 and 2010, the authors diagnosed that 88% of the fires occurred in the Amazon and Cerrado biomes. These researchers as well as others in the literature [50,51,52] say that regardless environmentalists and many experts disagree with this old practice of burning and demonstrate the severity of its environmental impact, this anthropogenic activity is still widely used mainly in in the Cerrado and Amazon as a way of clearing the soil and occupying new areas for agriculture or livestock. Unfortunately, this practice often hits forests and ends up becoming unmanageable [53]. This impact becomes even worse if we think the Amazon forest is responsible for retaining about 40% of all remaining tropical forests in the world [18] and most of the Legal Amazon area is in Brazil [51].

#### 4.1.2. State

Subsequently, the same technique was applied with the intention of identifying the States in which there was a greater incidence of fires. Brazil is composed of 26 States and 1 Federal District. For that, the geographic coordinates of fire outbreaks, fire risk, and expected precipitation, number of days without rain, identification of the Cities were used in the decision tree classification method. The results and the classification presenting the most striking States (red rectangle) are in Table 2.

For the Classification by States, it was obtained the identification of those who had the highest fire rates in the year 2017, being the state of Pará, Mato Grosso, and Maranhão. The classification by states using in the Weka software took 8.81 s to train the model and 0.84 s for testing, generating an overall accuracy of 99.39%. The Kappa statistic also indicates the very good concordance, as presented in Table 2. For the three States identified in Table 2, we applied the decision tree classification in the corresponding databases to interpret and identify climatic conditions and periods of the year where the number of fires becomes significantly high. This stage is fundamental to know the periods and characteristics where the burn rate is most significant. For that, the identification of date was removed from the database and the variables Day without Rain, Precipitation, Fire Risk, Latitude and Longitude were used in the classification. The error matrix for the months with the largest amounts of burns considering the three identified States are presented in Table 3. In Table 3, as already mentioned not all 26 Brazilian States are included, since the intention of this classification was to identify which States presented an excessive quantity of fires. Considering the whole dataset, the States of Pará, Mato Grosso and Maranhão together represent 51.35% of all fire observations recorded in 2017. Melo et al. (2011), in their study, had already identified that the regions of the Center-West and North have a greater susceptibility of risks of future fires, with September being the month in which the largest amount of fires occurs due to agroforestry, especially in the States of Pará and Maranhão. According to the Government of the State of Mato Grosso (2019), the use of fires in the period from July to October to clean rural areas is considered a crime, with a prison sentence and consequently payment of a fine. The Government also intends, in a second moment, to raise awareness and guide the farmers through campaigns on the harmful effects of burnings for nature and human health, focusing mainly on a prohibitive tone. This period was stipulated for the reason of the drought period, with low rainfall, facilitating the prospection of the fire and making it uncontrollable.

#### 4.1.3. Months, Climatic Conditions and Period of the Year

The next step was to apply the classification techniques in the databases corresponding to the three States identified in Table 3 to interpret and identify climatic conditions and periods of the year where the number of fires becomes significantly high. This stage is fundamental to know the periods and characteristics where the burn rate is most significant. First, the months in which the largest amounts of fires occur in the three identified states are analyzed, according to Table 3.

Analyzing the error matrix in Table 3, it can be observed that with a hit rate of 81.21%, the period of greatest incidence of fires comprises the period from July to December. The Kappa index was 0.7552, indicating also a high degree of agreement. The processing time needed to construct the training model required 40.46 s, while the time spent to test the model (Table 3) required only 0.42 s. Considering that 93.90% of all the outbreaks of fires occurred in 2017 were from July to December, a correlation analysis between fires and diseases in this period is of very relevant.

A similar period with burn-decease association was found in the work of [26], however only one municipality of the State of Mato Grosso and one year were considered.

These results are also in agreement with [54] indicating that the regions of the Center-West and North have a greater susceptibility to the risks of future fires, being September, the month in which the largest amount of fires occurs due to agroforestry mainly in the States of Pará and Maranhão. After discovering the months with high numbers of fires, a new categorical variable was created in the database, where the period from January to June represents the rainy season, and the period from July to December as a period of drought. With this, the classification was again generated in order to identify the relationship between the fire hazard, predicted precipitation and Rainless Days with the rainy and dry period classes. Because the variable Month was used to generate the new variable Period, it was eliminated from this analysis. The results of the classification by period in the testing set are presented in Table 4.

As can be observed, the hit rate of the error matrix presented a value of 94.24%, with the drought period being the most critical for fire outbreak. Other researches have shown that although diverse fire outbreaks are observed during the year reflecting in several climatic effects, they are more significant in the periods of drought [30,52,55].

This is also an important aspect to be taken into consideration for later analysis with diseases, mainly those related to the respiratory system. Because much more fire occurrences in the dry period, the confusion matrix was very unbalanced (Table 4), implying a very low Kappa index (0.0433), not necessarily reflecting low rates of overall agreement [56]. Regarding to the processing time, the training model took 1.86 s while the time spent to test the model took only 0.16 s.

**Figure 2:**
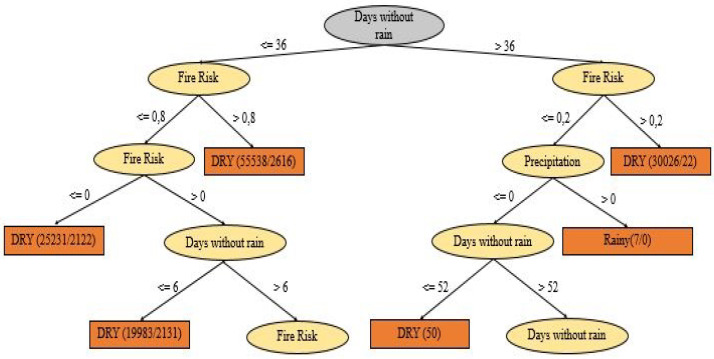
Representation of the Decision-Tree.

In order to better understand the relationship among the attributes fire risk, number of days without rain and predicted precipitation with the defined periods, Figure 2 presents the tree of decision with the partition conditions.

When analyzing the decision tree in Figure 2, two conditions can be observed regarding the classification of the periods:

(1) if the number of days without rainfall is less than or equal to 36 and if the fire risk is greater than 0.8 then a total of 55,538 fire outbreaks are correctly classified;
(2) if the number of days without rain is greater than 36, and if the fire risk is greater than 0.2 then a total of 30,026 fire outbreaks are correctly classified.

Furthermore, we can observe the decision tree (Figure 2) show how the other variables present in the database behave for a classification by periods as well as their order of importance. Also it is possible to predict situations favorable to fires by analyzing the decision tree generated without the geographic coordinates.

### 4.2. Clustering

After the stages of classification, where the states and periods were identified, the K-means algorithm was used to identify the groups of cities within each State for posterior quantitative analysis with diseases according to methodological flowchart in Figure 1.

As observed in Table 2, This analysis is going to be presented for the three states with the highest burn occurrences in the year 2017: Maranhão, Mato Grosso, and Pará.

### 4.3. Maranhão

**Figure 3:**
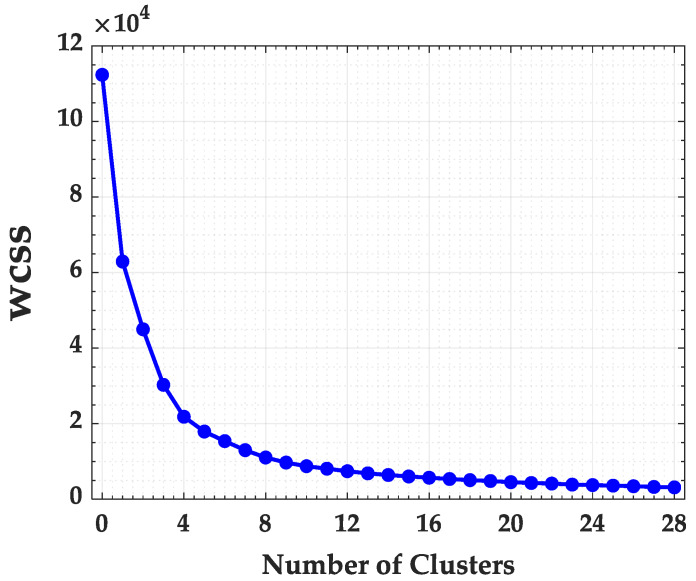
Within Cluster Sum of Squares (WCSS) for Maranhão State.

To better identify the optimal number of clusters for the State of Maranhão, the K-means and the Elbow method algorithms were used. In Figure 3 the Elbow method is presented considering the Within Cluster Sum of Errors (WCSS).

**Figure 4:**
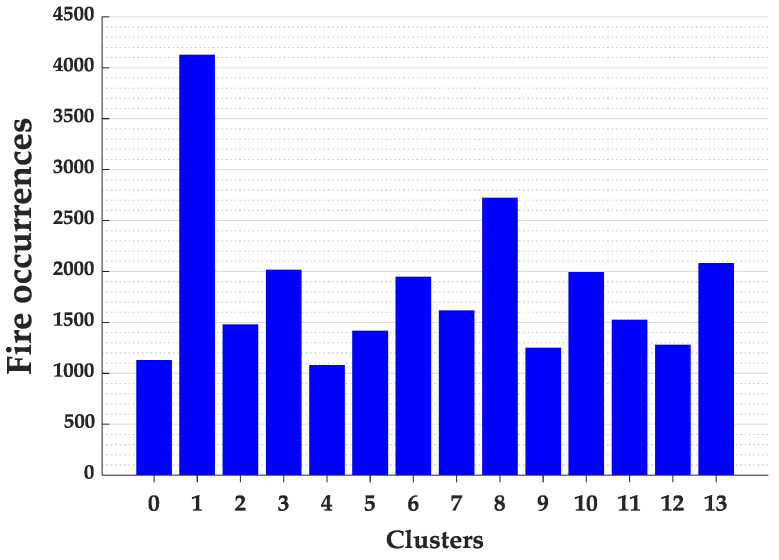
Fire occurrences for each cluster of the Maranhão State.

The application of the K-means algorithm and the Elbow Method in the State of Maranhão required 1 min and 3 s. Analyzing the Elbow method in Figure 3, it can be observed that the curve stabilizes from the cluster number of 14. Thus, the state of Maranhão was divided in 14 groups, as presented in Figure 4.

By dividing the State of Maranhão into 14 parts, according to Figure 4, it can be identified that cluster 1 presented a high value of fires in comparison to the other clusters. Thus, the cities in the cluster 1 were used to investigate the relationship of fire outbreaks with diseases of the respiratory system and other possible outbreaks of disease.

**Figure 5:**
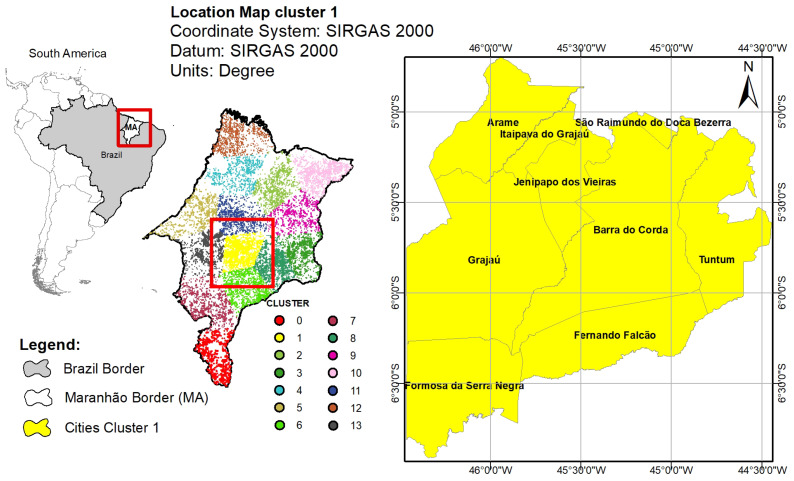
Cluster Distribution for the state of Maranhão.

The clustering of the cities is displayed in Figure 5. The yellow area represents the cluster 1, i.e., the group of cities where the largest number of fires occurred for the case of Maranhão State.

**Figure 6:**
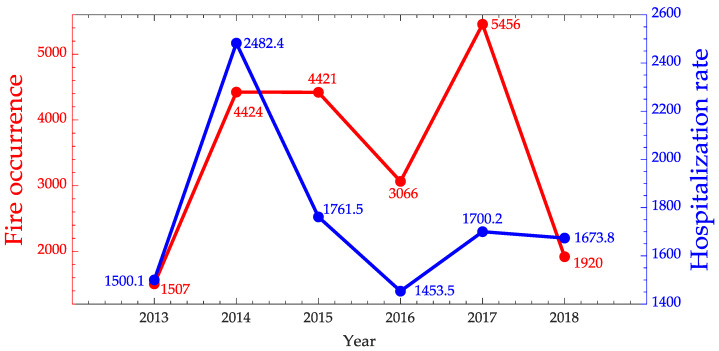
Time series of fire occurrences in red and hospitalization rate due to respiratory system diseases for children 0–4 years old in blue from July to December for Maranhão State.

**Figure 7:**
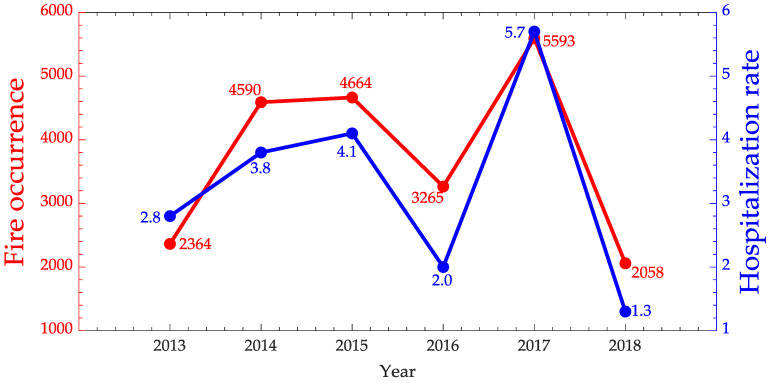
Time series of fire occurrences in red and Malaria hospitalization rate in blue for Maranhão State.

The time series of fire occurrences and hospitalization rates for diseases of the respiratory system in children 0–4 years old from July to December are represented in Figure 6 and Figure 7. From this figure and considering the Spearman coefficient was 0.66, the correlation between the hospitalizations due to diseases of the respiratory system and fire occurrences can be considered strong [47]. This result comes in the direction of the worry and importance of gaining a better understanding of spatial and temporal patterns related to respiratory health problems, as it is a growing cause of hospital morbidity [57].

Thus, this investigation is also important for other outbreaks, such as malaria. Analyzing Figure 7, it can again be observed that the fire occurrences in the last 6 years is directly related to malaria hospitalization rate, with an indication of a very strong correlation (Spearman coefficient of 0.94).

### 4.4. Pará

**Figure 8:**
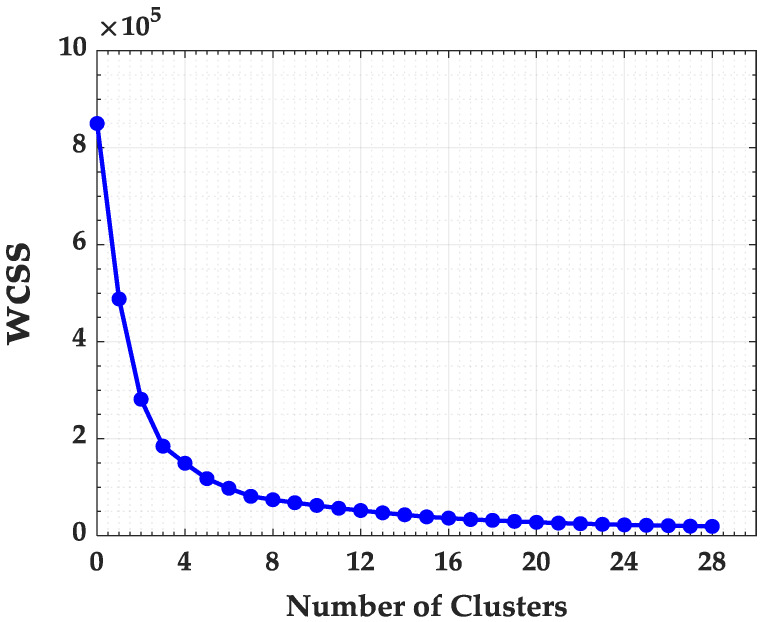
Within Cluster Sum of Squares (WCSS) for Pará State.

Pará State presented 61,729 focus of fires in 2017, being the state the largest number of occurrences. Again, to better identify the ideal number of clusters for the State of Pará, the K-means and the Elbow method algorithms were used. In Figure 8 the Elbow method is presented considering the within cluster sum of errors (WCSS).

**Figure 9:**
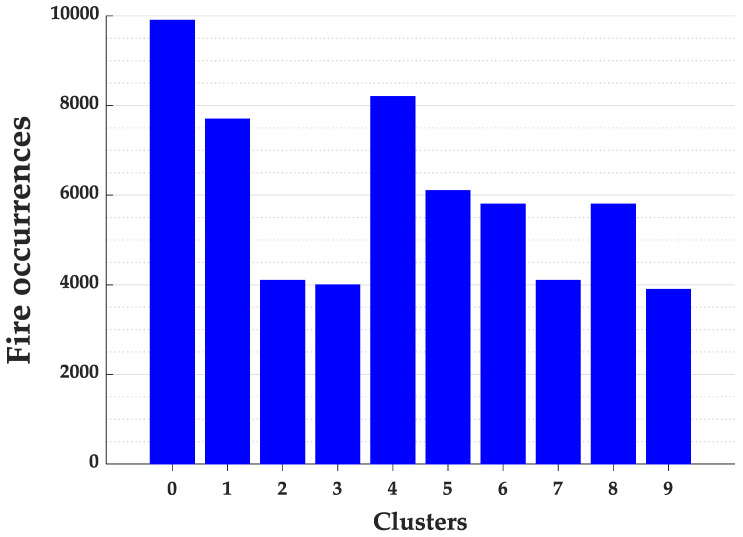
Fire occurrences for each cluster of the Pará State.

The processing of the Elbow algorithm to identify the ideal number of clusters in the state of Pará required 2 min and 14 s. Analyzing graphically the behavior of the sum of the square errors in Figure 8 we identified the ideal number of clusters equal to 10. The distribution of fire focus in the state of Pará for the 10 clusters are presented in Figure 9.

We can see in Figure 9 that the clusters 0 and 4 presented the highest frequency of fires. This behavior and the position of the centroids were kept even increasing the number of clusters, indicating the stability of these group formations.

**Figure 10:**
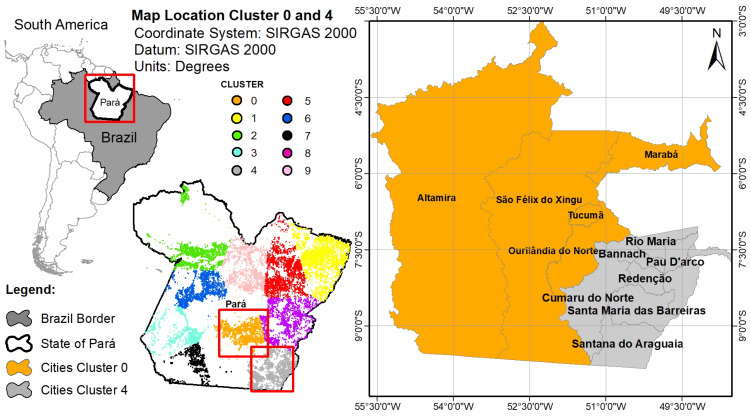
Cluster Distribution for the state of Pará.

Because to the highest frequency and proximity of clusters 0 and 4, the analysis of the number of fires with disease outbreaks will be performed together explicating the cities corresponding to each cluster (Figure 10).

In the map represented in Figure 10, the orange region represents cluster 0 and the gray region represents cluster 4. With the cities identified for each cluster in Figure 10, we investigated the temporal behavior of annual fire focus and hospitalizations for diseases of respiratory system, Malaria and Leishmaniosis in the cities from 2011 to 2018. Because the proximity of the clusters 0 and 4, numbers of fire focus in the cities of both clusters were aggregated for this temporal analysis with diseases.

**Figure 11:**
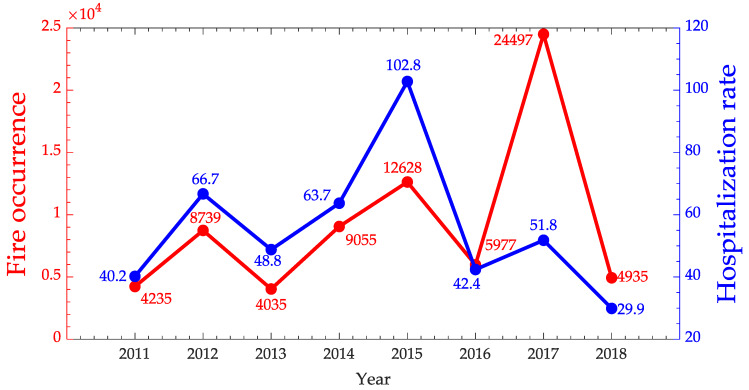
Time series of fire occurrences in red and Malaria/Leishmaniasis hospitalization rate in blue for Pará State.

The correlation of hospitalizations for diseases of the respiratory system and the burns for the cities identified in cluster 0 and 4 in the State of Pará was very weak (<0.19), but the respective one considering hospitalizations for malaria and leishmaniasis was much better ($r_{s}=0.7$), indicating a strong relationship between these variables. The time series of fire occurrences and hospitalization rate of Malaria and Leishmaniosis for clusters 0 and 4 can be visualized in Figure 11.

**Figure 12:**
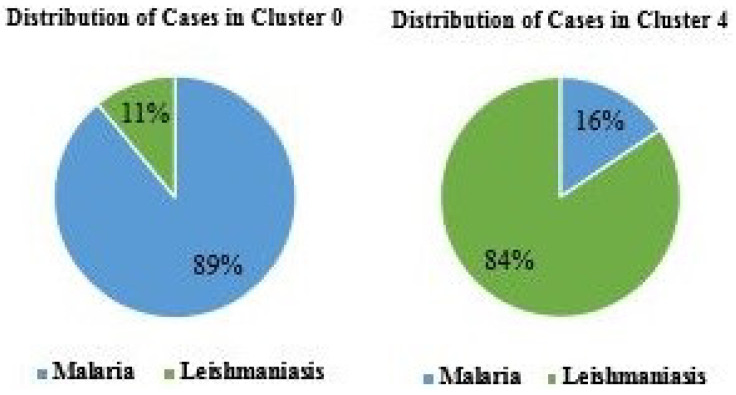
Proportion of Malaria and Leishmaniosis hospitalizations in the clusters 0 and 4 of state of Pará.

In the previous analysis, the Malaria and Leishmaniosis hospitalizations as well as the clusters 0 and 4 were aggregated, however, it may be important to investigate the behavior of them separately, as in Figure 12.

When analyzing the clusters individually, it was possible to identify a difference in the behavior of the diseases in each cluster. In the investigated period, 89% of all cases that occurred in cluster 0 represent malaria cases (Figure 6) while 84% of the cases of Leishmaniosis were in cluster 4.

Spatial heterogeneity in the incidence of Leishmaniosis is a fundamental aspect to be considered for planning disease control actions. Focusing interventions in areas considered to be at high risk could be a strategy to improve the effectiveness of the control measures and reduction of hospital costs of the single health system [58].

In a study carried out by [58], it was possible to correlate environmental issues related to deforestation with cases of leishmaniasis, but the results were not satisfactory for the identification of high-risk areas, being considered by the author a complex challenge. For [59], each every 10% increase without deforestation provides a 3.3% increase in malaria incidence mainly in the states of the Amazon region. This deforestation occurs through human behavior where economic development is attributed as justification. On the other hand, it is possible there are feedbacks between environmental change and health in a locally specific and context dependent.

Each region identified in the Data Mining can be studied separately, since each group of cities generated a disease outbreak that can be better analyzed by health specialists or researchers, in order to understand why a certain region tends to favor the occurrence of malaria cases and another to leishmaniasis. This fact that different regions exhibit specific characteristics of certain diseases has already been stated in the literature, such as in [60].

### 4.5. Mato Grosso

**Figure 13:**
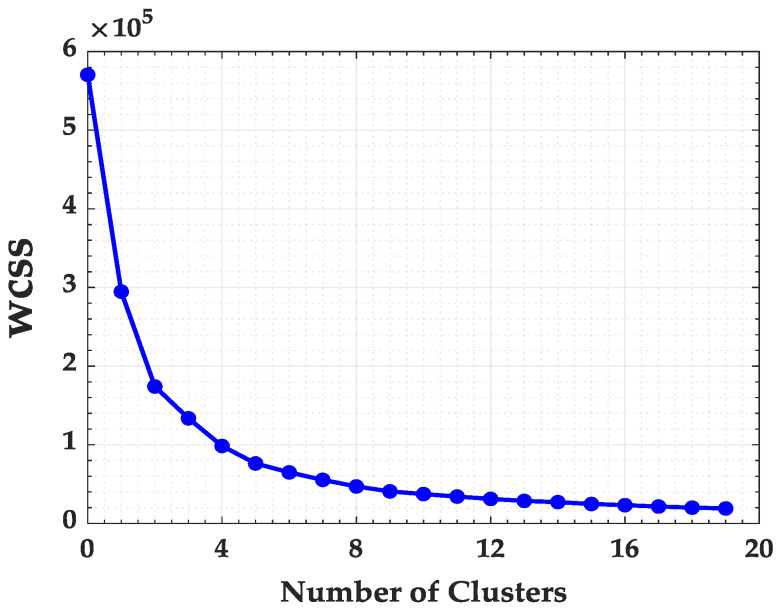
Within Cluster Sum of Squares (WCSS) for Mato Grosso State.

Again, following the methodology of the work, it was necessary to identify the ideal number of clusters for the State of Mato Grosso using the Elbow method, according to Figure 13.

**Figure 14:**
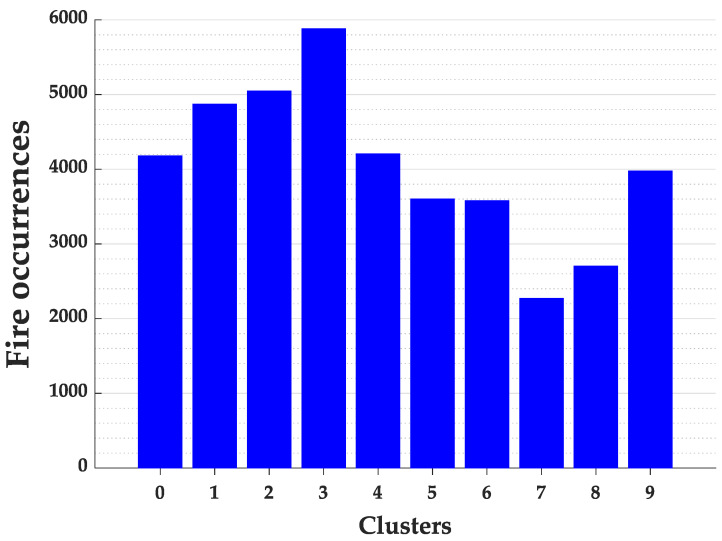
Fire occurrences for each cluster of the Mato Grosso State.

The time needed to process the K-means algorithm with the Elbow method required only 1 min and 15 s to identify the ideal number of clusters for the State of Mato Grosso. After identifying the ideal number of clusters, which was k=10, the frequency of fire focus by clusters can be seem in the histogram in Figure 14.

**Figure 15:**
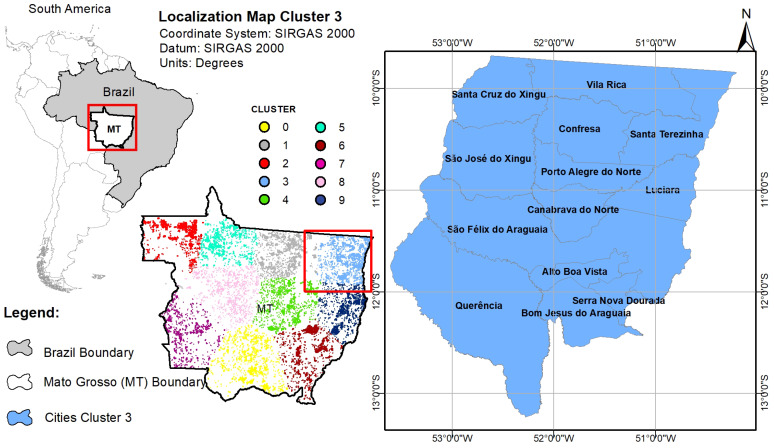
Cluster Distribution for the state of Mato Grosso.

As it can be identified in Figure 14, cluster number 3 represents the group of cities with the highest incidence of fires. Then, a map was created to represent and locate the cities belonging to this cluster according to Figure 15.

**Figure 16:**
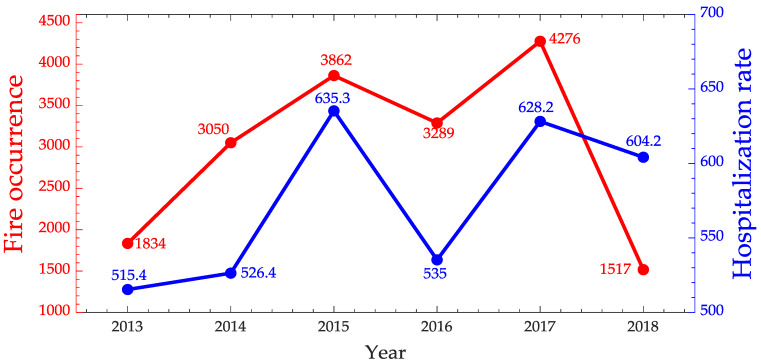
Time series of fire occurrences in red and hospitalization rate due to respiratory system diseases for children 0–4 years old in blue from July to December for Mato Grosso State.

Figure 15 is representing the distribution of clusters identified in the State of Mato Grosso. The location of cluster 3 as well as the cities belonging to it are also described. Once the cities were identified, the data were aggregated to build the annual time series of fire occurrences and the hospitalizations for respiratory diseases for children from 0 to 4 years old Figure 16 from July to December.

When analyzing Figure 16 we can notice an association between burnings and hospitalizations due to diseases of the respiratory system for children 0–4 years old for the period from July to December. The Spearman correlation indicates a strong association of 0.6. Considering most of the fire focus occur in this period, it reinforces the hypothesis that the impact of the emission of gases expelled by fires may be directly affecting this age group. However, no relationship was found between fires and hospitalizations for malaria and leishmaniasis in cluster 3 belonging to the State of Mato Grosso ($r_{s}\leq 0.19$).

## 5. Final Remarks

From Data Mining it was possible to use geographic observations to identify regions with high occurrence of fires. Instead of losing a lot of time to verify the consistency of a database, the application of the classification techniques allowed to identify states, biomes, seasons of the year and climatic characteristics with great success rates. All the error matrices presented accuracy rates above 93% and the processing time to generate them was generally less than 10 s, which is a great result considering a database with more than 260 thousand records of fire outbreaks. From the classification, the states of Maranhão, Pará and Mato Grosso, which are in the edges of North, Northeast and Central-West Brazilian regions, and dry season presented the high occurrences of fires.

With clustering methods, specifically, the K-means algorithm, it was possible to identify the groups of cities with the highest occurrences of fires within each state for the quantitative analysis with diseases (Figure 1). The processing time from the performed implementation for this step could be considered very good as well, once it took about 2 min. With the identification of small groups of cities, it was possible to identify regions with a very strong temporal correlation between fire focus and hospitalizations due to diseases of the respiratory system, malaria and leishmaniasis. Each region showed a susceptible behavior to some disease as well as some degree of correlation with fire focus. It was detected that the highest occurrences of fires was from July to December, period used for analysis of respiratory diseases. The cluster identified in the State of Maranhão presented a strong relationship between fires and diseases of the respiratory system for children 0–4 years (Spearman correlation $r_{s}=0.66$) and very strong when considered malaria hospitalizations ($r_{s}=0.94$). In the state of Pará, the unique relationship found was with hospitalizations of malaria and leishmaniasis ($r_{s}=0.7$). For the state of Mato Grosso, it was detected correlation only between fire focus and diseases of the respiratory system ($r_{s}=0.6$). Furthermore, we could verify that even close regions (neighbor clusters) can show more susceptibility to different diseases. This was evident to the state of Pará regarding to hospitalizations of malaria and leishmaniasis. Hence, generalizing results found in this work for the entire state, Meso-region, Microregion or simply to a city, may be a mistake due to the diverse characteristics of each region.

Thus, spatial heterogeneity is a fundamental aspect to be considered for planning disease control actions, specially in the incidence of leishmaniosis and malaria. Focusing interventions in areas considered to be at high risk could be a strategy to improve the effectiveness of the control measures and reduction of hospital costs of the single health system. This natural spatial variability where different regions exhibit specific characteristics of certain diseases reinforces the importance of studying each region separately. On the other hand, such study would be really difficult to be carried out for so many localised regions, but becomes fully feasible with the methodology presented in this research, where the critical regions were identified in voluminous databases from Data Mining and associations with diseases could be performed.

Finally, it is important to highlight that the presented method allows reliable and fast extraction of useful information from massive and complex spatial/geographic and temporal data reducing the chance of errors or misleading conclusions.

The capability of analysing such large datasets are very important mainly because of the growth of community health information. These agile and effective models can promote the interest of public agencies in order to create policies to control the proliferation of diseases, and consequently, reduce abusive costs with treatments of diseases that can be reduced or even avoided having the knowledge of the specific regions where there is greater incidence. Accurate analysis can benefit early disease detection and understanding. This understanding can assist in patient care with the provided community services.

Besides the reached results, this research just came to contribute as a method to overcome the aforementioned lack of localized analyses for the problem in question as well as other areas.

In further investigations, other point event cluster approaches can be applied and compared. Anisotropic methods would be interesting as well since in K-means points in each cluster are modeled as lying within a sphere around the cluster centroid. Indeed, it is important to consider other factors, such as temperature, relative humidity and expected precipitation that may contribute to describe and predict the local characteristic of each specific region and the inherent spatial variability.

## Figures and Tables

**Table 1:** Error Matrix of the classification per biome in the testing dataset.

a	b	c	d	e	f	← Classified as:
**304**	0	0	0	0	0	a = Pampa
0	**3732**	0	105	22	0	b = Caatinga
0	0	**39,641**	117	0	0	c = Amazônia
0	121	202	**25,637**	110	30	d = Cerrado
15	26	0	119	**5662**	0	e = Mata Atlântica
0	0	3	10	0	**2176**	f = Pantanal
Correctly Classified Instances				77,152		98.8609%
Incorrectly Classified Instances				889		1.1391%
Kappa statistic				0.9816		
Mean absolute error				0.0058		
Root mean squared error				0.0569		
Relative absolute error						2.8282%
Root relative squared error						17.7031%
Total Number of Instances				78041		

**Table 2:** Error Matrix for State Classification in the testing dataset.

a	b	c	d	e	f	g	← Classified as:
**737**	0	0	0	0	0	21	a = Rio Grande do Sul
0	**2497**	0	0	0	0	18	b = Bahia
0	0	**1102**	0	0	0	0	c = Ceara
0	0	0	**18,514**	4	26	0	d = Pará
0	0	0	0	**12,118**	0	0	e = Mato Grosso
0	0	0	6	0	**9446**	0	f = Maranhão
0	11	0	0	0	0	**3184**	g = Minas Gerais
...	...	...	...	...	...	...	n =...
Correctly Classified Instances					77,563		99.3875%
Incorrectly Classified Instances					478		0.6125%
Kappa statistic					0.9931		
Mean absolute error					0.0007		
Root mean squared error					0.019		
Relative absolute error							0.9965%
Root relative squared error							10.4955%
Total Number of Instances					78041		

**Table 3:** Error Matrix of Month Classification in testing set.

a	b	c	d	e	f	g	h	i	j	k	l	← Classified as:
**86**	23	29	8	5	3	3	2	12	29	43	24	a = Jan
11	**39**	26	7	4	5	1	1	0	15	20	9	b = Feb
17	23	**87**	28	2	0	0	5	1	35	17	6	c = Mar
15	12	34	**86**	16	2	0	5	15	29	24	4	d = Apr
16	3	10	20	**150**	33	11	10	27	63	19	9	e = May
11	0	1	0	41	**751**	60	49	88	58	22	14	f = Jun
2	4	1	0	2	53	**2486**	186	140	55	106	37	g = Jul
4	0	2	2	7	20	206	**5499**	519	179	132	50	h = Aug
2	1	1	4	11	48	121	468	**14956**	354	174	64	i = Sep
19	8	33	24	17	56	45	205	612	**3984**	398	117	j = Oct
25	15	19	20	6	16	91	128	146	429	**2871**	268	k = Nov
21	5	15	9	6	11	27	56	87	169	363	**1662**	l = Dec
Correctly Classified Instances										32,657		81.2101%
Incorrectly Classified Instances										7556		18.7899%
Kappa statistic										0.7552		
Mean absolute error										0.0371		
Root mean squared error										0.1624		
Relative absolute error												28.8298%
Root relative squared error												64.0739%
Total Number of Instances										40213		

**Table 4:** Error Matrix for Period Classification in the testing dataset.

a	b	← Classified as:	
**58**	2276	a = RAINY	
39	**37,840**	b = DRY	
Correctly Classified Instances		37,898	94.2432%
Incorrectly Classified Instances		2315	5.7568%
Kappa statistic		0.0433	
Mean absolute error		0.2286	
Root mean squared error		0.2286	
Relative absolute error			95.4071%
Root relative squared error			97.7459%
Total Number of Instances		40213	
